# Listening to the Voices of Mothers Who Participated in a Video Feedback Intervention for Postpartum Depression

**DOI:** 10.1177/23333936241245588

**Published:** 2024-04-15

**Authors:** Jennifer Bon Bernard, Nancy Moules, Suzanne Tough, Panagiota Tryphonopoulos, Nicole Letourneau

**Affiliations:** 1 University of Calgary, AB, Canada; 2 University of Western Ontario, London, Canada

**Keywords:** postpartum depression symptoms, hermeneutics, video-feedback intervention, mothering, Canada

## Abstract

Postpartum depression (PPD) symptoms can negatively influence mother-infant interactions. Video-Feedback Interaction Guidance for Improving Interactions Between Depressed Mothers and their Infants (VID-KIDS) is a parenting intervention that allows mothers experiencing PPD symptoms to observe and improve their interactions with their infants. VID-KIDS has also positively influenced infants’ stress (cortisol) patterns. There is limited research on maternal perspectives of interventions like VID-KIDS. In this hermeneutic study, four mothers were interviewed to increase understanding of the VID-KIDS experience. Key findings included: 1) VID-KIDS provided an opportunity for mothers with PPD symptoms to positively transform their identity; 2) VID-KIDS provided a chance to witness the mother-infant relationship forming and improve maternal mental health t, and; 3) VID-KIDS provided a space for mothers to dialogue about their experience with PPD symptoms authentically. VID-KIDS promoted healing from PPD as mothers experienced a transformation in how they perceived themselves and their relationships with their infants.

Postpartum depression (PPD) is a significant public health concern (Almond, 2009) that affects between 17.22 (Wang et al., 2021) to 23% of mothers (StatCan, 2023). Described as the “thief that robs mothers of the love and happiness they expected to feel toward their newborn babies” (Beck, 2002, p. 453), PPD interferes with mother-infant interactions (Field, 1995; Mueller et al., 2019), primarily due to the symptoms (e.g. fatigue, low mood) that function to decrease maternal sensitivity (Beck, 1995; Slomian et al., 2019). When parental sensitivity diminishes, infants’ stress levels increase (Lawler et al., 2019; Rattaz et al., 2022), with potentially negative influences on cognitive and social-emotional growth in children (Finegood et al., 2017; N. Letourneau et al., 2011; Rattaz et al., 2022).

Implementing interventions aimed to improve mother-infant interactions should be a priority when designing programs for women suffering from postpartum depression. One such mother-infant intervention is called Video-Feedback Interaction Guidance for Improving Interactions Between Depressed Mothers and Infants (VID-KIDS; Tryphonopoulos & Letourneau, 2020). It was developed in 2014 to improve the relationship between mothers with postpartum depressive symptoms, and their infants. VID-KIDS involves registered nurses (RNs) providing feedback to likely depressed mothers after viewing a video of the mother and infant playing together, using materials and concepts underpinning the Parent-Child Interaction Teaching Scale (PCITS; Oxford & Finlay, 2015). The Parent-Child Interaction Teaching Scale is an extensively researched, reliable, and valid tool to measure the quality of mother-infant interactions (Oxford & Finlay, 2015). Tryphonopoulos and Letourneau (2020) pilot tested VID-KIDS to evaluate its effects among 12 mothers with depressive symptoms, on the following outcomes: 1) mother-infant interaction quality, 2) infant cortisol patterns, 3) infant development, 4) depression and anxiety in the postpartum period, and 5) parenting stress. Their pilot study revealed that VID-KIDS significantly improved mother-infant interactions and infant cortisol patterns (Tryphonopoulos & Letourneau, 2020).

While the pilot study demonstrated considerable promise, mothers’ views of VID-KIDS are unknown. In general, there is limited research on understanding mothers’ perspectives related to video-feedback intervention. While recommended (N. L. Letourneau et al., 2017), opportunities for mother-infant dyads to engage in treatment together are currently uncommon, indicating that more evidence about this type of intervention is needed (Kornaros et al., 2019).

## Description of the VID-KIDS Study

VID-KIDS is a video-feedback parenting intervention designed to support mothers experiencing PPD symptoms to interact with their infants responsively and sensitively. The program is appropriate for infants from 2 to 8 months of age. The intervention is conducted in a series of three home visits over 15 weeks. Sessions are between 60 and 90 minutes in duration, beginning with the RN providing education related to interpreting infant cues. This educational component involves the RN reviewing flashcards depicting infants displaying both engaging and disengaging cues. Next, the mother selects an activity from a list provided with the Parent-Child Interaction Teaching Scale (e.g., teach her infant to shake a rattle or pass a block from one hand to the other) that she can use to interact with her infant while being videotaped. The mother is informed that her role is to teach her infant how to do the activity, and she is videotaped for a maximum of 5 minutes. The RN and mother co-view the video 3 times. The first viewing does not involve any discussion between the RN and mother. The second viewing consists of a conversation related to engagement and disengagement cues. The third viewing consists of highlighting instances of the serve and return relationship. Serve and return refers to the back and forth interaction between a child and caregiver when cues are responded to with sensitivity (Center on the Developing Child Harvard University, n.d.; Komanchuk et al., 2023). The feedback involves using a strengths-based approach, based on the Parent-Child Interaction Teaching Scale assessment guidelines for a healthy mother-infant interaction. The RN also provides up to 30 minutes of social support (i.e. informational, affirmational, emotional) (Cohen et al., 2000; Stewart, 1993) related to the mother’s experience with PPD, as a foundation to the intervention. For example, if a mother is distressed about being unable to afford food or experiencing limited social support, the RN can offer guidance about appropriate resources, such as local food banks or community resource centres, to help address these needs. Addressing such needs prior to the onset of the intervention focused on maternal-infant interaction quality is thought to be essential for mothers to be able to benefit from the interaction guidance.

## Research Question

To summarize, the aim of this study was to understand maternal perspectives about participating in VID-KIDS. How mothers understand their relationships with themselves, their infant and nurse, during the experience of PPD and the VIDS-KIDS intervention has not been explored. Thus, the research question for this study was: What are mothers’ lived experiences of the VID-KIDS intervention?

## Method

This research project was guided by hermeneutics, commonly referred to as “interpretive inquiry” (Moules et al., 2015, p. 2). Hermeneutics is a research method articulated by Moules et al. (2015), that is guided by a philosophical grounding in the work of Hans-Georg Gadamer. The approach aims for understanding rather than explanation, using interpretation to help illuminate situations/topics in which we encounter meanings that are not immediately understandable (Gadamer, 2008). This research applied hermeneutics to derive meaning from what the mothers shared about their experience participating in the VID-KIDS intervention, and in applying an *interpretative effort*, different and rich understanding was sought.

## Participants

In this study, the researcher and the first author (JB) recruited four mothers who had completed participation in VID-KIDS. This was an adequate number of mothers to interview as in qualitative research, the deep exploration that is done case by case, can result in a uniquely rich understandings of the experience under study (Sandelowski, 1995). The inclusion criteria included completing the VID-KIDS intervention within the last 6 months and a score above 12 on the Edinburgh Postnatal Depression Scale (EPDS) screening tool, as administered by RNs at the infant’s immunization clinic. EPDS scores above 12 are frequently consistent with physician diagnosis of major depressive disorder in the postpartum period (Georgiopoulos et al., 2001). The primary researcher (and the first author; JB) of this hermeneutic study was the RN who delivered the VID-KIDS intervention. Filling this dual role offered benefits from the pre-established rapport between JB and the mother, which promoted mothers’ comfort in the interview. JB purposely invited mothers to participate based on their potential to be articulate and provide meaningful, rich data for analysis. Mothers were also invited who demonstrated inquisitiveness about the intervention, as in hermeneutics, this is thought to increase the likelihood of obtaining a deeper understanding (Moules et al., 2015). Mothers were excluded if they were less than 18 years of age and non-fluent in English. Using these criteria, seven mothers met the inclusion criteria, of which four mothers consented and completed the interview. Follow-up or validation interviews were deemed unnecessary due to the richness and completeness of data collected.

## Data Collection

During the process of informed consent, ethically approved by the University of Calgary Conjoint Health Research Ethics Board (REB18-1985), mothers agreed to be audio-recorded during interviews. Hermeneutic interviews involve more than merely requesting participants to describe their experiences, but rather the researcher aims to understand what matters most to participants while keeping with the topic under investigation (Moules et al., 2015). Recordings were transcribed by JB using audio playback and typed verbatim into MS Word. Transcripts were kept on a secure server to which only JB had access.

Six pre-determined questions guided the interviews such as: How might you describe your experience of being involved in the VID-KIDS study? What was the biggest impact and perhaps the biggest disappointment, if any? How do you think the VID-KIDS intervention might be improved? And what would be important to conserve in it? With discernment and sensitivity, JB had the freedom to ask different questions that invited new meaning and where different understandings would emerge. Maintaining flexibility in the conversation resulted in the length of the interviews varying from one to two and a half hours. According to Gadamer (2008), when fluidity is preserved in interviews, the researcher’s understanding leads the partners in dialogue to discover new insights and unexpected findings. In other words, the conversational nature of interviews in hermeneutics creates a dialogue that allows the other to be heard (Gadamer, 2008). The interviews were audio-recorded in the mothers’ homes, which provided convenience and comfort. While data saturation was not directly assessed, JB and the co-authors determined that the interviews resulted in sufficiently rich and meaningful data for analysis and was well-suited to answer the research question.

## Data Analysis

The first author (JB) read and reflected upon the mothers’ interviews repeatedly until understanding and new knowledge was discovered. Data that were interpreted to be the most compelling, that is, those that “catch our attention and call for a destruction of meaning” (Moules et al., 2015, p. 118) were the focus. As the aim of this study was to arrive at a “different” meaning or understanding, coding the data and searching for themes was not a goal (Moules et al., 2015). Rather, to arrive at interpretations in hermeneutics, “researchers read for ideas that stand out, raise questions, provoke curiosity, answer questions, catch our attention and most importantly, call our present understanding into question” (Moules et al., 2015, p. 126). The curiosity that is sparked in the analysis, directs the hermeneutic researcher to dig into relevant literature to look for a fresh angle on the topic, guided by the data (Moules et al., 2015). Participant quotes are shared that provide an opportunity to reach a new interpretation, related to the phenomenon of interest (Moules et al., 2015).

## Findings

The participants were Caucasian mothers over the age of 30 years, with post-secondary education, household incomes over $60,000, and were either married or in significant relationships with the infants’ fathers. EPDS scores improved for all four mothers from the beginning of the study to the end, with two mothers changing their thoughts of “wanting to harm herself” to “no feelings of wanting to harm herself.” Parent-child interaction quality also improved for all four mothers. All infants were full-term and healthy at birth. In hermeneutic inquiry, the findings often include relevant literature to support interpretations (Moules et al., 2015). In this way, the writing style differs from other qualitative approaches, that typically distinguish the results from the discussion. In this section, therefore, we present nine interpretations, grounded with participant quotes in Table 1 and in text, along with accompanying literature. Given that only four mothers were interviewed, information identifying particular mothers being quoted was not included to help guarantee anonymity.

**Table 1.** Meanings and Participants’ Quotes.

Meaning	Quotes from four mothers
1. In Witnessing, Transformation Emerged	*I felt like even though I felt sad, she didn’t feel that, like even in my dark moments, it is like she wasn’t being dragged down with me. I was definitely worried that I was dragging the whole house down with me but looking at those videos I was like no, it is actually okay—there isn’t some horrible dark cloud over this entire house . . . I am not actually screwing up I didn’t feel like I was . . . failing that badly.* *When I am watching the video, it feels like I am . . . doing something right because she is happy and you don’t have that feeling of anxiety that everything you do is harming . . . So when I am watching the video and she is happy . . . it makes me feel like okay . . . we are doing good things together . . . you just don’t feel hopeless . . . you are actually doing what you are supposed to be doing as a parent and you are not letting the anxiety or the anger or the dark thoughts . . . creep in to make you second guess yourself.*
2. Finding the Power to Get Out of the Fog	*I felt she was very engaged with me and I think that made me realize that I need to take more time to have those moments with her. . . I think seeing those moments made me want to engage with her more. Seeing her like light up and hearing her laugh and like her trying to copy what I am doing is incredible . . . It made me excited to engage with her.* *I never realized how much she watches me. It matters what I am doing and what I am saying and I guess it made me feel more motivated to pull myself out of the fog and put energy into my mental health so that I can be there with them and not just . . . give up. I would say that I didn’t feel strong but I knew that I could be and I didn’t feel like I had hope but I knew that I needed it. So like watching the videos of me looking at her, looking at me, I was like . . . she needs a strong Mom . . . I can be the strong Mom, I can be this good Mom . . . I can be a good Mom right now and I can still work towards being better.*
3. Understanding Roots of Disempowerment	*The . . . nurse that diagnosed me at the . . . clinic was a real B [for slang derogatory term “Bitch”]. She basically . . . checked me off and then . . . called my doctor and was like either you are making an appointment to go and see your doctor or I am calling her, ‘cause someone needs to see you . . . she basically . . . strong-armed me and said . . . ‘cause you are not okay . . . And I know they just . . . crank people through because they are busy or whatever but . . . those little questionnaires, every time since that, they have asked me to fill out I have been . . . no thank you—I am declining . . . I have been diagnosed by your little system and I am getting help elsewhere . . . I don’t know how honest people are checking off those lists. This is the very first time that I was like going to lie on the check boxes and then I was like no I feel terrible-maybe I should just check the boxes and maybe someone will help me and I did and then she acted horrible . . . she acted like I was . . . a naughty kid-like I had done something wrong.*
4. The Healing Power of Connection	*I think for a couple of days I would try to keep that interaction going and try to find times where we could just do that together, so that she could have that again because I saw how much she really enjoyed that and it does make you feel good that you might be doing something right as a parent, that you can enjoy something that simple . . . that happy feeling probably like a day or two at least, so it was nice. It definitively helped me . . . feel less trapped because you get to feel that excitement again and you feel like . . . we can make this a regular occurrence.*
5. Understanding the Necessary Beauty: The Mother-Infant Relationship	*With depression, I feel like sometimes you don’t think that you are doing the best job or that the kid doesn’t really like you and you don’t really like them so I think when someone else is telling you that oh she is really engaging with you . . . it is . . . very helpful. It kind of makes you realize oh we do have a relationship. We are forming a relationship . . . she is looking at me, she is telling me she is interested, she is telling me that she wants to participate in this activity. Before I participated in this study, I was just . . . taking care of this little person and I really didn’t think that there was a relationship there . . . I thought I was just taking care of this blob . . . it just kind of allowed me to deepen my relationship with her.*
6. The Relational Flow—Getting Harmony in Return	*I guess it just feels natural-it feels like I am not forcing it. I don’t really think about it, like I am happy, this is what I want to do . . . like I am not doing it on purpose I guess. I am just happy and I just want to engage with her and I want to make her laugh and I want to make her smile . . . I never really said, I am going to sing her a song, we are just engaging with another and previously you know that is the response that I have gotten and I know when I sing to her it will make her happy . . . I think that focusing in on the fact that there is a serve and return relationship and that you know what you put out is going to bring something back to you.* *I don’t know with being in the red zone, probably my anxiety, just not being so uptight about everything and just being more laid back I guess with him, and kind of giving him more freedom, because I couldn’t let him do anything. I always had to be right there hovering all the time.*
7. Understanding the Infant as a Competent Healer	*I think that it is crazy how much they can tell you without being able to talk.* *I think it definitely has a positive impact knowing that I can teach her things—it has been a very positive impact on my well-being. . . knowing that I can make her feel good and that she wants to engage with me. I feel like as the engagement increases . . . as she has more expression, has more ability to move her hands and her feet and her face and everything. I feel like that this has improved our relationship and it has improved my happiness—I feel like we are starting to understand each other better . . . I felt like she could if I took enough time she could do anything.*
8. A Call for Authenticity	*I feel like when you are in your home it is more real-like you can have a more authentic experience-whereas when you are in a clinical setting it kind of feels forced . . . Whereas, if we went somewhere else she might be distracted by . . . different things and she wouldn’t be able to engage the same way that she does at home. Whereas, if you were to take her to a clinic, you know it might be like carpeted floor, white walls and fluorescent lighting and I feel like you know she would need some time to take all that surrounding in before she was able to properly authentically engage with me, whereas here she is comfortable.*
9. Authenticity Invites Curiosity	*I guess just you asking questions too, because if you just left it open ended I wouldn’t be able to talk but a lot of questions you ask, I was like okay that was a good question and it made me think . . . deeper. Whereas, if it was more like one ended it would be like okay talk about your experience, I would just be like blah, blah and that is all I would say but you had a lot of these questions where I was okay I can obviously reciprocate and give back to you—so questions that I never even thought of . . . so that is why I don’t like really surveys because they are always . . . so closed . . .. it is always like bleep, bleep.*
10. Being Real in Dialogue—The Back and Forth Matters	*Knowing that you will not be judged for what you say or . . . what you see-like you didn’t come in and think that my kids were gross or my house was yucky or . . . turn your nose up to anything or say I wish you would have done this better-there was no judgment at all. You told me about your kids—yeah—this was some good back and forth so it wasn’t just me on the spotlight by myself -having to put it all out there-which is easier to talk when it is not just that.*

### Meaning 1: In Witnessing, Transformation Emerged

VID-KIDS offered mothers an opportunity to powerfully witness their capabilities related to mothering, informing them that they were flourishing in *becoming*, or redefining themselves as a mother. For one mother, when she observed the interaction with her infant, it was a moment that provided her with confidence and significant relief upon ascertaining that there was not a “horrible cloud over the house” and that she was not “screwing up.” This mother’s interpretation of her secure connection with her infant provided a new meaning related to how she moved in the world. Her renewed understanding that she was “doing something right” allowed for a re-definition of herself as a mother. With this awareness, maternal strength and a new sense of self emerged. As a result of this wisdom, feelings of affirmation emerged that provided maternal restoration and rejuvenation. Her healing was grounded in the realization that she was “doing what she was supposed to be doing.” This finding aligns with observations in another qualitative study in which mothers expressed that “viewing from the outside” (Vik & Hafting, 2009, p. 291) provided opportunities to acknowledge that they were succeeding as mothers.

Mothers with mental health concerns have expressed distress that others will judge them as not being a worthy parent (Blegen et al., 2010; Knudson-Martin & Silverstein, 2009). As a result, there is persistent pressure bestowed on mothers to ensure that others perceive them as competent. PPD has been described as an expected response to the societal expectations that encapsulate the serious and often stressful demands of motherhood (Berggren-Clive, 1998). In the formidable world of mothering, to be told you are doing a job well-done is the ultimate badge of honor. Mothers with mental illness experience challenges related to preserving their identity and explained that there is limited support available to understand this struggle (Shor & Moreh-Kremer, 2016). Abrams and Curran (2011) highlighted that when mothers’ experiencing PPD are confronted with additional social strain, such as financial issues, developing a positive identity becomes increasingly complicated. Indeed, “shattered role identity” has been described as the initial stage mothers encounter with PPD (Chen et al., 2006, p. 450).

How can we provide compassion for mothers who are in the process of distinguishing their new role as a mother when experiencing PPD? Mercer (2004) recommended using the word becoming, instead of the word attainment, in her theorizing about the process of identifying as a new mother. This reframing provides a more accurate representation of the fluid growth pattern in motherhood that many women experience. This term welcomes vulnerability, and in turn, striving to be a perfect mother naturally subsides. Becoming is related to growing, metamorphosis, turning, and never-ending (Merriam-Webster, n.d.b). In comparison, the word attaining is associated with scoring, winning, beating, and excelling (Merriam-Webster, n.d.a)

VID-KIDS provided mothers with a supportive space where they could *become* a new mother, without facing the pressure of *attaining* the status of a perfect mother. As Gadamer (1960/2013) stated, “If we thus regard experience in terms of its result, we have ignored the fact that experience is a process” (p. 361). For this mother, in her process of learning about her mothering from a different perspective, strength became unleashed, and she understood herself as powerful and wise.

### Meaning 2: Finding the Power to Get Out of the Fog

From the mothers’ voices in VID-KIDS, after observing their connection with their infants, they developed a refreshed sense of self that contributed to an increased desire to make positive changes. One mother felt “motivated to pull herself out of the fog” after she understood how much she mattered to her infant. This new expansion provided this mother with the capacity to act to improve her mental health. For another mother, when she became aware of how much her infant engaged with her, she felt inspired to have more similar interactions. In this instance, she felt enabled to take action, once she understood how much she meant to her infant. In this moment of awe, her maternal power was unveiled.

This discernment of how mothers with PPD symptoms may experience empowerment is meaningful because in the presence of mental illness, self-assurance as a parent decreases (Perera et al., 2014; Reck et al., 2012). Vik and Hafting (2009) found similar findings where mothers were filled with confidence and motivation to make affirmative changes in their way of being after observing themselves interact with their infants. Additionally, researchers have stressed the importance of providing support that fosters maternal competence for mothers experiencing PPD (Arante et al., 2020).

Gadamer (1960/2013) informed us that “we always find ourselves within a situation, and throwing light on it is a task that is never entirely finished” (p. 312). VID-KIDS *threw light* on how important it was for the mothers to observe their interactions with her infant. Motherhood is a *situation* where growth and learning are continuous for both the mother and infant. To support mothers in maintaining a healthy trajectory of growth, it is important for health care professionals to highlight their capabilities and ensure their successes shine-just as in the experience of VID-KIDS.

### Meaning 3: Understanding Roots of Disempowerment

In exploring the roots of powerlessness that persevere in the experience of PPD symptoms, it is valuable to examine systemic contributions to this persistent anguish that mothers face. How does societal discourse contribute to the disempowerment of mothers, impede witnessing her wisdom, and silence her voice? One mother described being “strong-armed” and told she needed to be seen by someone right away because she was “not okay.” She shared that her diagnosis by the “little system” gave her a sense of regret, and unfortunately, in her state of vulnerability, her perception was that she was scolded. She described feeling like she was “a naughty kid” and “doing something wrong” in this moment of insight. Perhaps, this is a situation that calls for what Byrne (1999) referred to as “dismantling professional boundaries” (p. 69) and recommended that for this to happen, there needs to be movement from caring *for* to caring *with*. This mother’s experience of powerlessness came from being desperately honest and experiencing unintended negative consequences as a result. This type of support did not align with how she envisioned the nurse would be *with* her in this instance of vulnerability.

Disharmony between the health care professional and mother may stem from how mental illness is constructed and perceived in health care and society (Savvidou et al., 2003). Traditionally, in health care, with good intentions, there is an emphasis on evaluating mothers’ performance, which is amplified when a mother is experiencing a mental illness (Savvidou et al., 2003). Furthermore, from a societal context, maternal capabilities remain hidden and there is a tendency toward identifying weakness over strengths, which Savvidou et al. (2003) described as “deficit-based discourses” (p. 399). Additionally, health care professionals control the diagnosis and treatment plan, which can be associated with feelings of powerlessness and difficulty making sense of one’s illness (Benaroyo & Widdershoven, 2004). This approach can be discomforting, as determining the most appropriate health care goals is optimally achieved with a collaborative conversation between the health care professional and patient (Epstein et al., 2005).

When providing support to mothers experiencing PPD it is necessary to provide respectful and collaborative health care, focused on understanding mothers’ suffering and offering compassion (Dennis & Chung-Lee, 2006). How would the experience shift for mothers if the identification of their competencies and capabilities became the primary focus? If maternal wisdom were honored, the discord between the health care professional and mother would perhaps subside and a compassionate connection would emerge. In VID-KIDS, maternal strength is acknowledged, and mothers can find some happiness.

### Meaning 4: The Healing Power of Connection

In observing themselves interacting with their infants on the video playback, mothers reported a medicinal type of connection, that infused throughout the body, comparable to the way an IV rapidly infuses drugs. The medicine was observing the positive mother-infant interaction, and the effect was pure authentic “happiness” and “excitement” that would last “for one day or two days.” This infusion of joy seemed to be the “treatment” that one mother reported needing. Similarly, in another study, the authors explored the parental experience of viewing themselves interact with their infant (Gill et al., 2019) with one parent describing this experience to be comparable to receiving a “vitamin injection” that was filled with “new insight, coping experience and empowerment” (Gill et al., 2019, p. 6).

Although mothers expressed that biological mechanisms could cause depression and a diagnosis provided a sense of reprieve, it is important to understand how social experiences may offer insight into causes (Gammell & Stoppard, 1999). Shaikh and Kauppi (2015) described the importance of being open to many different biological to socio-political explanations of PPD, and recommended that we “need to honour the interpretations of clients, counter the hegemonic influence of the medical model and provide services that align with clients’ worldviews” (p. 475). Furthermore, when implementing a medical linear treatment pathway, there is a risk that contextual causes of PPD may not be recognized (Montgomery et al., 2006; Schreiber & Hartrick, 2002), resulting in a limited understanding of the experience, contributing to difficulty in healing.

Given the vast array of maternal concerns regarding PPD treatment (Guo et al., 2020) and differing perspectives on PPD causes (Pearlstein et al., 2009), it is vital to think about novel ways health care professionals can deliver care. VID-KIDS is an innovative intervention that is accepted by mothers and has the potential to increase happiness without adverse side effects, stigma, and guilt. This finding suggests that VID-KIDS can be wisely implemented in health care as a complementary form of treatment for PPD with the traditional biomedical interface for PPD, in collaboration with the mother. VID-KIDS illustrated a compelling association between maternal healing and the witnessing of the mother-infant connection.

### Meaning 5: Understanding the Necessary Beauty: The Mother-Infant Relationship

Universally, the mother-infant relationship is the initial opening for love and connection (Bergum & Dossetor, 2005). Mothers living with a mental illness have such a profound yearning to experience connection with their children and have indicated that this is so imperative they created coping mechanisms such as “masking” (Montgomery et al., 2006, p. 24) to hide their suffering, in an effort to preserve this relationship. Therefore, given how necessary the formation of a relationship is for mothers in the face of mental illness, it was an illuminating moment for one mother when she observed that she was no longer looking after a “blob” and that there was indeed a relationship forming. This finding parallels Vik and Hafting (2009), finding that mothers need to “discover” their child to participate in mutual interactions with them. (p. 292).

The Latin word for relationship is *necessitudio*, which means “necessity” (WordHippo, n.d.b) and *forma* is the Latin word for form, which is described as “beauty” (WordHippo, n.d.a). When these two words are combined, the forming of a relationship can be interpreted as a necessary beauty. The revelation for this mother, that a relationship was forming, was a moment that provided her with relief and clarity. This mother was reassured that a deep connection with her infant was established, and it was in this instance she understood her *necessary beauty*.

How does a mother know in her heart that a relationship is forming? One mother interpreted that a relationship was established after witnessing the non-verbal communication of her infant “looking” and “telling” her that she was interested in participating, which transpired during her engagement in the VID-KIDS intervention. In this moment, when the RN and mother exchanged knowledge related to the infant’s active involvement in their interaction, she was filled with sudden relief that a relationship had taken shape.

How do health care professionals engage in conversation related to understanding how mothers perceive their relationship with their infant in the experience of PPD symptoms? VID-KIDS provides experiential learning, observation, and dialogue with mothers that explores these perceptions. For an infant to actively contribute to relationship building, the freedom to explore is essential. When there is freedom for exploration, the mother and infant have an increased likelihood of experiencing a positive relationship.

### Meaning 6: The Relational Flow—Getting Harmony in Return

Promoting optimal mother-infant interaction quality or “flow” between the mother and her infant is of primary importance in VID-KIDS. One mother communicated that when she was involved in serve and return moments with her infant, there was a “natural” feeling, and she was “not forcing it.” Another mother shared when she offered more “freedom” and less “hovering,” her baby spent less time in the red zone. In the red zone, infants display signs of emotional distress, and the communication flow is disrupted. When the mother-infant dyad is flowing, shared *harmony* is visibly pronounced.

As human beings, we experience the state of flow “when all the contents of consciousness are in harmony . . .” (Csikszentmihalyi & Csikszentmihalyi, 1988, p. 24). Similarly, Vik and Hafting (2006) described the communication in a healthy mother-infant connection as a “harmonized relation” (p. 237). How does this harmonious concept of flow protect mothers from PPD symptoms? For humans to experience the state of flow, focus and presence are required, which minimizes distressed thoughts (Csikszentmihalyi & Csikszentmihalyi, 1988). This stress-free zone highlights the importance for mothers to engage in moments of flow with their infants, as a strategy to improve happiness.

In looking at the inverse of this interpretation, how does impaired flow (or interaction) between a mother and infant manifest? The opposite of flow would suggest what Barr (2008) referred to as “mechanical infant caring” (p. 366), which is how mothers related to their infants when they felt disengaged. From a societal lens, rationalization was raised as a potential hindrance toward the movement of flow because it prefers control over spontaneity (Csikszentmihalyi & Csikszentmihalyi, 1988). In the context of the mother-infant relationship, the need to be *rational* might be an outcome of the numerous parenting books and the overwhelming advice on social media related to being the perfect mother. This exemplifies how public discourse in parenting might be associated with maternal desire to follow the rules, expectations, and standards. As a result, this type of societal dialogue can potentially negatively impact the flow in the mother-infant relationship.

Health care professionals must evaluate and promote the quality of mother-child interaction because of the potential connection it has in improving maternal mental health (Adina et al., 2022). Utilizing measures such as the Parent-Child Interaction Teaching Scale, used in the VID-KIDS study affords the opportunity for mothers and infants to learn how to experience optimal interactions or flow that can create a state of connectedness and happiness. In turn, when infants interact in a relationship that respects freedom and spontaneity, the infant’s perceptive proficiencies may be uncovered.

### Meaning 7: Understanding the Infant as a Competent Healer

One mother described her elation in her infant’s cleverness when she stated, “I think it is crazy how much they can tell you without being able to talk.” She went onto say that her “infant could do anything” if she provided sufficient time. The revelation of her infant’s profound competency to connect despite not engaging verbally in dialogue caught this mother off-guard. The non-verbal communication provided a level of protection for this mother in that her depressive symptoms improved when she understood her infant’s insightfulness.

Infants are gifted with a profound perceptiveness that contributes to their success as social communicators (Goldberg, 1977; Stern, 1977). Stern (1977) acknowledged that infants arrive with formidable capabilities to establish relationships (p. 33). During the VID-KIDS intervention, infants illustrated this capability. The English word capable means “sufficiently able, having power or capacity,” and is derived from the French word *capable*, which means “able, sufficient; able to hold” and the Latin word *capabilis*, which means “receptive; able to grasp hold” (Online Etymology Dictionary, n.d.b). This can be interpreted that infants have the power to *hold* their mother *sufficiently* in a state of contentment, therefore illustrating a capacity to protect her from unhappiness. This example of infant capacity is an essential finding because when mothers are experiencing postpartum depression they can have difficulty “holding onto their infants” (Arante et al., 2020, p. 22).

Furthermore, when a mother interprets her infant to be socially capable, her self-regard is elevated (McGrath et al., 1993). As one mother shared, her happiness improved with an increase in her infant’s expression and engagement. Gill et al. (2019) used the term “mutuality” (p. 5) to describe the interrelated bond that parents maintain with their children. The discovery of this profound interconnection during the video viewing led to improved energy and expressiveness between the parents and the infants (Gill et al., 2019).

### Meaning 8: A Call for Authenticity

Mothers value physical and emotional environments that provide comforting space for their authentic way of being to unfold. Authenticity is rooted in the Greek word “authentikos” which means “original, genuine, principle,” and stems from “authentes” which means “one acting on one’s own authority (Online Etymology Dictionary, n.d.a). This etymology of authenticity aligns with the way in which this mother was wanting to feel like she could be her “own” in her home. To further support a mother’s desire to maintain her authentic way of being, Dubus’ study explored the maternal experience of a relational-based postpartum intervention and a theory was developed entitled “permission to be authentic” (Dubus, 2014, p. 43). In the context of receiving parenting support, this type of engagement was deemed to be of central importance to the mothers (Dubus, 2014).

One mother chose to use the word “forced” to describe the experience of what it would be like to interact with her infant in a clinical setting. She explained that you can have a more “authentic experience when you are in your home.” The opposite of force is natural, effortless, and easy. It is within the confines of an unforced environment where authenticity resides. A state of authenticity is challenging for individuals to experience when freedom is not preserved (Freire, 2000). Unfortunately, mothers with mental illness find it challenging to experience authenticity because they are faced with the tension of trying to hide their anguish, while at the same time searching for the freedom to be honest (Montgomery et al., 2006). Evidence has revealed that mothers silence their voices when experiencing PPD symptoms (Knudson-Martin & Silverstein, 2009; Mauthner, 1998) Knudson-Martin and Silverstein (2009) determined that mothers with PPD symptoms endured feelings of incompetency and disconnection with others, which further escalated their isolation.

To thrive and be *one’s own*, clinical care settings must strive to build spaces that mimic a home away from home. A home away from home is an environment that promotes openness where mothers feel they can *truly* express their stories of vulnerability and suffering. What type of relational environment is needed to ensure that mothers can move with freedom and comfort to express their voice in a way that feels “real?” How does a mother experiencing PPD symptoms find her place or home in clinical settings? One way is by providing care that values open and heartfelt dialogue, and as Bergum and Dossetor (2005) stated, “the call to relationship is a call to dialogue” (p. 220).

### Meaning 9: Authenticity Invites Curiosity

One mother described preferring questions that she had “never thought of before” because they made her think “deeper.” She valued the reciprocal conversation between the RN and herself during the VID-KIDS intervention. The element of curiosity brought forward in an authentic conversation plays a central role in the unfolding of understanding. This mother appreciated inquiry as an invitation to have her voice heard in a meaningful manner. Additionally, this mother valued questions that supported openness to explore difference, and there was a welcomeness to entering into a potentially unknown territory of her experience. Gadamer (1960/2013) describes the value of asking a question or questionability, as bringing into the open the fact that an answer is not decided and remains undetermined. Experience filled with *questionability* reminds us that, “being curious involves being aware that the situation is not as tidy as it might seem” and that during distressful experiences, such curiosity may protect mental well-being (Epstein, 2017, p. 47). Encounters between the RN and mother reflect the value in preserving inquisitive exploration and the importance of curiosity when implementing parenting interventions for mothers who have symptoms of PPD.

### Meaning 10: Being Real in Dialogue—The Back and Forth Matters

One mother elucidated that she appreciated the sharing of personal information from the RN as it meant she would “not be the only one in the spotlight.” Pieranunzi (1997) found that when nurses went “beyond the surface, beyond the “patientness,” of the person to the person” (p.160), their engagement became more meaningful and genuine with patients. There was a plea from this mother to escape the feeling of *patientness.* She desired a human-to-human interaction versus an RN-to-mother conversation. With softened boundaries, she described it as “easier to talk,” thus increasing the chances of the power between the RN and the mother becoming equalized. When power is equalized, dialogue and understanding can expand in a more natural and fathomable way. Fenwick et al. (2001) found *“*chatting” (p. 583) between the RN and mother contributed to improved maternal self-esteem and infant bonding.

In interpreting the experience of authenticity, understandings related to the taken-for-granted moments in dialogue for mothers experiencing PPD symptoms are uncovered. Through the words of the mothers, the everydayness and subtle moments in conversation are perhaps the big moments that pave the way to authenticity. VID-KIDS shows how interpersonal exchanges do not have to be elaborate to be meaningful, but rather, they need to be genuine and compassionate.

## Summary and Implications

This research aimed to understand maternal perspectives about participating in VID-KIDS. The mothers in this study provided persuasive evidence that VID-KIDS is beneficial, acceptable, and, most importantly, healing amidst the experience of symptoms of PPD. Three key findings included: 1) VID-KIDS provided an opportunity for mothers to view themselves differently (Meanings 1, 2, 3), 2) reflection on the establishment of the mother-infant relationship is of central importance (Meaning 2, 4, 5, 6, 7), and 3) it is imperative to provide an environment where mothers feel safe to be authentic about their experience with PPD (Meaning 3, 8, 9, 10).

VID-KIDS offered space for mothers to re-define their identity. When mothers observed their interactions, they beheld their capabilities in connecting with their infant. This discovery motivated mothers to take action to improve their mental health so that they could be meaningfully present when they engaged with their infant. Mothers also experienced an elevated sense of joy when learning how competent they were in their abilities to interact with their infants. Thus, mothers described an increase in maternal happiness, attributable to the mother-infant relationship, not medication or other therapeutics. The maternal reframing that was witnessed in VID-KIDS is a critical finding. To optimize healing in the context of mental illness, it is essential to achieve a positive self-perception (Yanos et al., 2010).

Second, in VID-KIDS, mothers reflected on the formation of the mother-infant relationship. In understanding that a relationship was indeed formed, there was maternal relief and reassurance. When mothers embraced and recognized their relationships with their infants, they witnessed decreased infant distress and experienced improved mental health. In addition, when mothers observed their interactions, they discovered their infants’ ability to communicate competently. This understanding of the infant’s capabilities contributed to further improvements in maternal mood.

Finally, mothers yearned to have their unspoken words about their experience with PPD verbalized, heard, and validated by the RN. This research revealed that the taken-for-granted elements of communication welcome authenticity and are important to preserve in VID-KIDS. From the voice of the mothers, an authentic relational connection, with the RN delivering the intervention should be established at the beginning and maintained throughout the intervention. This study revealed the need for health care professionals to bear witness to the experience of PPD symptoms in a non-judgmental and compassionate manner and is a component that is essential in the delivery of video-feedback interventions.

## Implications for Clinical Practice

This study's findings contributed to novel understanding about what is deemed to be of most importance in VID-KIDS from the mothers’ perspectives. Meaningful evidence was attained for incorporating VID-KIDS into clinical practice when mothers are experiencing symptoms of PPD. Findings suggest that the application of VID-KIDS’ strength-based approach offers an opportunity to understand maternal and infant interactive competencies that may in turn increase maternal mental health. VID-KIDS was also revealed to be an innovative intervention for the management of PPD symptoms, that has the potential to complement traditional medical treatment. Exploring alternative treatments for PPD symptoms is important because desirability for medication is a concern postpartum for mothers (Pearlstein et al., 2009). Further, mothers valued the collaborative participation in VID-KIDS, compared to more traditional medical treatment that may be more one-directional. As mothers regarded genuine dialogue with the RN to be an essential component of VID-KIDS, this should be a priority in this and similar interventions. Finally, the delivery of VID-KIDS in the home environment offered several benefits, including decreasing barriers associated with accessing PPD support and increasing the likelihood of authentic engagement.

## Limitations and Strengths

This study was the first to explore maternal perceptions related to VID-KIDS and only one of five studies that examine mothers’ perspectives of video-feedback interventions (Gill et al., 2019; Vik & Braten, 2009; Vik & Hafting, 2006, 2009). A benefit of this study was that the RN who delivered the intervention conducted the interviews. This relationship contributed to mothers feeling comfortable to share their thoughts openly. Following the principles of hermeneutics, the direction of this interview focused on what mattered most to this topic from the mother’s perspective, resulting in a richer understanding. With the application of hermeneutics, some of the complexities and tensions associated with the experience of postpartum depression were uncovered, which may not have been revealed with research methodology that applies a more structured interview process.

Limitations of this study might be that it is not representative of the diversity associated with PPD. Although not a goal with hermeneutic research, the lack of sample variability may raise a concern related to transferability. Although seen as a benefit and critical in hermeneutics, other research traditions may raise concerns related to social desirability bias, in that given the same individual delivered the intervention and conducted this research, the mothers may have been reluctant to provide a sincere answer. Self selection and recall bias may also have been operating. Future research could involve conducting more interviews to continue to expand understanding with varying demographic factors considered, and considering the utility of broader categories of health care professionals, beyond RNs, delivering VID-KIDS (e.g. social workers, psychologists).

## Conclusion

VID-KIDS promoted healing for mothers experiencing symptoms of PPD. Mothers reported experienced a transformation in how they perceived themselves as mothers, their relationship with their infant, and their encounter with PPD symptoms. Their changed beliefs emerged from combining the science of VID-KIDS with a supportive environment that allowed their wisdom to flourish, resulting in a profound *becoming.* This new-found awareness put mothers in a position to move more confidentially and reassuringly in the world. It was the mothers’ perceptive ability to interpret themselves as competent interactive partner with their infant, that provided healing. In summary, “like everything meaningful, the beautiful is einleuchted [defined as clearly evident, shining in]” (Gadamer, 1960/2013, p. 500). Participation in VID-KIDS was filled with beauty and meaning for the mother and infant-it was an *einleuchted* experience.
